# Mapping patient education encounters in elective surgery: a cohort study and cross-sectional survey

**DOI:** 10.1136/bmjoq-2024-002810

**Published:** 2024-05-27

**Authors:** James Booker, Jack Penn, Chan Hee Koh, Nicola Newall, David Rowland, Siddharth Sinha, John G Hanrahan, Simon C Williams, Parag Sayal, Hani J Marcus

**Affiliations:** 1 Victor Horsely Department of Neurosurgery, National Hospital for Neurology and Neurosurgery, London, UK; 2 Wellcome / EPSRC Centre for Interventional and Surgical Sciences, University College London, London, UK; 3 Queen Square Institute of Neurology, University College London, London, UK; 4 Neurosciences Department, Cleveland Clinic London, London, UK; 5 Department of Neurosurgery, The Royal London Hospital, London, UK

**Keywords:** Medical education, Process mapping, Surgery

## Abstract

**Objective:**

Develop a process map of when patients learn about their proposed surgery and what resources patients use to educate themselves.

**Design:**

A mixed methods design, combining semistructured stakeholder interviews, quantitative validation using electronic healthcare records (EHR) in a retrospective cohort and a cross-sectional patient survey.

**Setting:**

A single surgical centre in the UK.

**Participants:**

Fourteen members of the spinal multidisciplinary team were interviewed to develop the process map.

This process map was validated using the EHR of 50 patients undergoing elective spine surgery between January and June 2022. Postprocedure, feedback was gathered from 25 patient surveys to identify which resources they used to learn about their spinal procedure. Patients below the age of 18 or who received emergency surgery were excluded.

**Interventions:**

Elective spine surgery and patient questionnaires given postoperatively either on the ward or in follow-up clinic.

**Primary and secondary outcome measures:**

The primary outcome was the percentage of the study cohort that was present at encounters on the process map. Key timepoints were defined if >80% of patients were present. The secondary outcome was the percentage of the study cohort that used educational resources listed in the patient questionnaire.

**Results:**

There were 342 encounters which occurred across the cohort, with 16 discrete event categories identified. The initial surgical clinic (88%), anaesthetic preoperative assessment (96%) and admission for surgery (100%) were identified as key timepoints. Surveys identified that patients most used verbal information from their surgeon (100%) followed by written information from their surgeon (52%) and the internet (40%) to learn about their surgery.

**Conclusions:**

Process mapping is an effective method of illustrating the patient pathway. The initial surgical clinic, anaesthetic preoperative assessment and surgical admission are key timepoints where patients receive information. This has future implications for guiding patient education interventions to focus at key timepoints.

**WHAT IS ALREADY KNOWN ABOUT THIS TOPIC**

- Patient education is frequently overlooked preoperatively, which leads to misunderstandings regarding diagnosis, treatment and potential outcomes after surgery.

**WHAT THIS STUDY ADDS**

- Process mapping is an effective method for identifying when patients are seen by healthcare staff preoperatively and given information. The primary source of information used by patients to understand their impending surgery is verbal guidance provided by the surgeon.

**HOW THIS STUDY MIGHT AFFECT RESEARCH, PRACTICE OR POLICY**

- This study may guide future patient education interventions targeted at key timepoints along the process map.

## Introduction

Patient education is a crucial component of an integrated surgical pathway. Educating patients about their diagnosis empowers them to become an active member in shared decision making, improves treatment adherence and subsequent patient outcomes.^1 2^ However, patient education is often neglected which can lead to misunderstandings about diagnosis and treatment, and outcome. This is particularly true in elective surgery, where there can be a discrepancy between the surgical team’s intention and the patient’s expectation because the goals of surgery are often focused on improving quality of life by targeting specific symptoms. This impacts patients’ understanding of procedures which is notoriously poor in elective spinal surgery—the recall of general information 2 hours following a consent process is 18%, with only 65% of patients recalling more than two of six major risks of the operation.^3^ As a consequence, failure to warn patients of the risk of spinal surgery is a major cause of clinical negligence claims.^4^ Therefore, improving patient education in spine surgery is a key area for service quality improvement. Providing additional information to patients in the form of verbal explanations, information booklets, interactive websites and videos has been shown to improve information recall during consent.^5^

To improve patient education in spinal surgery, it is first vital to identify the key events within the patient pathway where information is presented.^6–9^ This can be achieved through process mapping, a methodology adapted from the engineering sector that visually represents the patient journey to identify points in the current system where interventions can be made to improve healthcare.^9 10^ Process mapping has largely been underused in healthcare; however, it has the potential to identify inefficacies and inadequacies in clinical care in current systems.^11^ Process mapping provides an opportunity to improve patient education in spinal surgery by identifying key timepoints through which a high percentage of patients pass through. Subsequently, these key timepoints can be targeted for interventions that aim to optimise patient education.

We propose that process mapping can be used as a useful tool in identifying what resources are used and where in the surgical process map patients are educated about their diagnosis and surgical management, using elective spinal surgery as an exemplar. The aims of this study were twofold. First, we aimed to develop a comprehensive process map of when patients learn about their proposed surgery. Second, we aimed to investigate how patients educate themselves about their upcoming surgery.

## Methods

### Study design and setting

A two-stage mixed methods study design combining qualitative development of the process map and quantitative validation using electronic healthcare records (EHR) was used. Following this, a cross-sectional patient questionnaire identified what educational resources patients use preoperatively. Development of the process map occurred from October 2022 to July 2023, at a single tertiary-academic centre in the UK.

This study developed an initial process map based on clinical experience from the authorship. The process map showed the elective spine surgery patient pathway prior to surgical intervention and illustrated the educational resources patients may use to learn about their surgery. The aim of the map was to identify key timepoints between the first clinic and surgical procedure where patients had encounters with healthcare staff. The initial process map was then further reviewed through semistructured interviews with key stakeholders. Key timepoints were identified at clinical steps of the process map when >80% of patients were present. The patient encounters in the process map were validated quantitatively using EHR.

**Figure 1:**
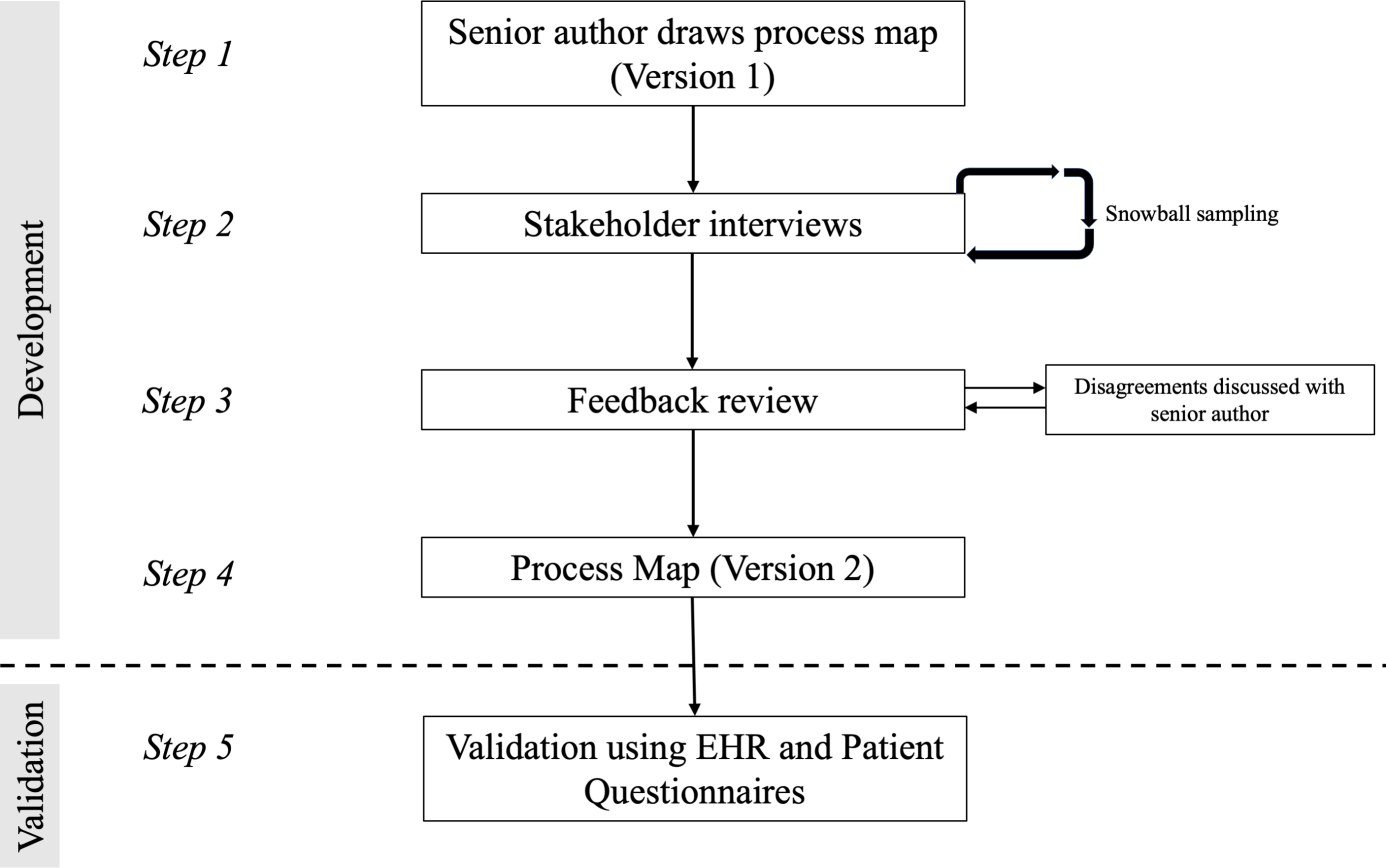
Study flow diagram.

The validated process map was then used to design a patient questionnaire which investigated the educational resources patients use (see figure 1).

### Development of the process map

The methodology used to develop the process map was adapted from Antonacci *et al* and used sequential phases of process map development until agreement was achieved.^11^ The process map that was developed focused on the period between the initial clinic appointment and the time of surgery.

During the first phase, the first (JP) and senior author (PS), who is an expert clinician in spinal surgery and the clinical lead for the specialty, drafted version 1 of the process map. This was followed by semistructured interviews with key stakeholders to review the process map. Stakeholders were defined as members of the clinical team that are present in spinal multidisciplinary team meetings. These included consultant surgeons, specialty registrars, consultant anaesthetists, clinical nurse specialists and physiotherapists. A snowball sampling methodology was also used, whereby stakeholders would make recommendations of other stakeholders who were subsequently interviewed. This process was stopped when no further stakeholders could be identified.

A five-part questionnaire (online supplemental file 1) was used to structure interviews and record qualitative information about the opinions and recommended changes that should be made to version 1. Additionally, an electronic copy of version 1 of the process map was displayed on a tablet which stakeholders could annotate on.

10.1136/bmjoq-2024-002810.supp1 Supplementary data

Feedback from the stakeholder interviewers was gathered and tabulated in a spreadsheet. The comments were reviewed by first authors (JB, JP), with relevant comments informing a redesign of version 1 process map to produce version 2. The changes made were reviewed and either accepted or rejected during research meetings with a consultant neurosurgeon (HJM) and head of complex spine surgery (PS).

### Validation of the process map

The process map was validated using a methodology described by Hanrahan *et al*.^12^ The EHR of patients that presented with degenerative spinal disease (DSD) and were managed surgically between January and July 2022, at a tertiary-academic single centre in the UK was used. A sample size of 50 patients was chosen as it was deemed adequate to gain an in-depth understanding of the patient pathway.^13^ Patients were identified through the EHR, EPIC (EPIC systems corporation, Wisconsin, USA) from the listed primary diagnosis of DSD with a procedure between January 2022 and June 2022. Patients below the age of 18 were excluded from the study.

Patient pathways were reviewed using EHR and the clinical encounters during the pathway after being offered elective surgery for DSD. This was then compared with the version 2 process map and the percentage of patients present at each clinical step of the process map was calculated. Key timepoints were defined as clinical steps at which >80% of patients were present. This was further divided into three groups—80–89%, 90–99% and 100%.^14^

### Investigation of educational resources

The educational resources that patients used to learn about their spinal procedure were identified prospectively between January and July 2023 at a single centre. Patient questionnaires were given either on the day of discharge following surgery or in postoperative follow-up clinic. This was a separate patient cohort to that used to validate the process map. This survey was developed with reference to a scoping review by Atlas *et al* which identified sources of information patients accessed prior to elective surgery (see online supplemental appendix B).^15^

### Patient and public involvement

While patients were not engaged in the initial design phase of this study, their direct involvement occurred postoperatively through a patient questionnaire. This survey sought their insights on the educational resources used to learn about their surgery. Their responses provided valuable perspectives on the effectiveness and relevance of the educational materials, contributing to the study’s broader understanding of patient experiences.

### Study checklist

The Strobe checklist was used in guiding the write up of the manuscript.^16^

## Results

### Development of process map

**Figure 2:**
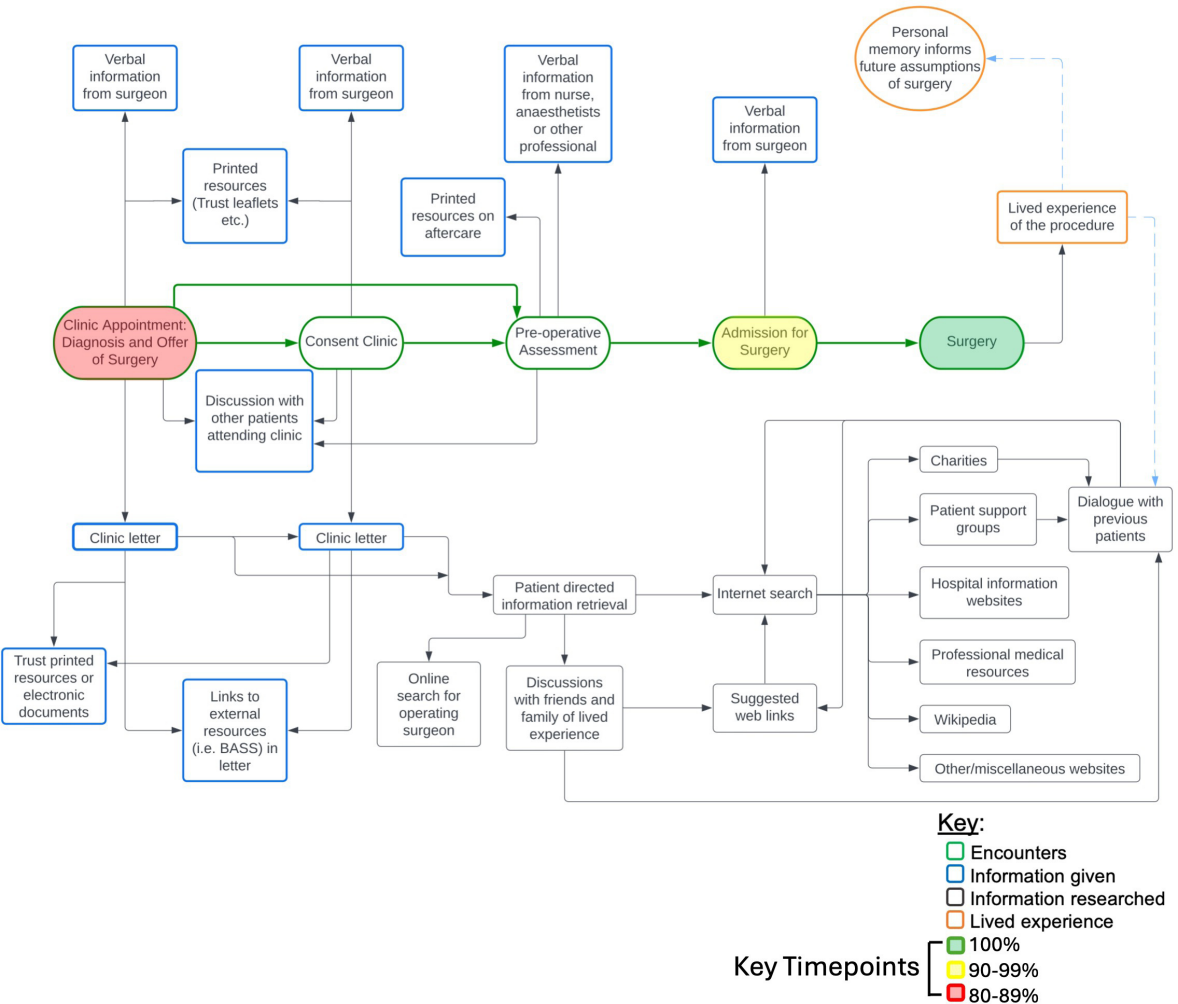
Elective spine surgery process map. This figure illustrates the pathway of encounters for patients undergoing elective spine surgery, a collaborative effort developed through semistructured interviews with 14 members of the spinal multidisciplinary team. Colour-shaded process steps indicate % presence in the real-world dataset: green=100%; yellow=90–99%; red=80–89%.

Version 1 of the process map, detailing the key timepoints that patients received information before undergoing surgery for DSD, was created by the authorship (see figure 2). The process map was used as a template for discussion during 14 stakeholder interviews. Stakeholders were mainly part of the surgical team (n=11), followed by anaesthetic team (n=2) and anaesthetic preoperative assessment nursing team (n=1). The most common stakeholders were consultant surgeons (n=4), followed by consultant anaesthetists (n=2), clinical nurse specialists (n=2), surgical specialty registrars (n=4), physiotherapist (n=1) and preadmission coordinator (n=1). The total number of years of experience of the stakeholders in managing patients with DSD was 207 years with a median of 15 years (IQR=10–20).

From the stakeholder interviews, 34 recommended changes were internally discussed with the authorship. Of these, eight comments were rejected, five were not relevant to the process map and 21 were accepted by the authorship and informed changes to process map version 1 to make Version 2 (see figure 2).

### Validation of process map

Version 2 of the process map was quantitatively validated by reviewing the EHR of 50 patients who presented to a single centre with lumbar DSD from January 2022 to June 2022. Baseline characteristics of the patients are shown in table 1. No patients were excluded from the quantitative validation.

**Table 1:** Baseline characteristics

Characteristic	N=50*
Sex	
Female	28 (56%)
Male	22 (44%)
Age at procedure	62 (50, 69)
Operation type	
Cervical decompression	16 (32%)
Lumbar decompression	16 (32%)
Lumbar interbody fusion	10 (20%)
Other	8 (16%)
Number pre-procedure days after initial clinic	326 (98, 820)

*n (%); median (IQR).

Three hundred and forty-two encounters occurred across the cohort, with 16 discrete events identified. Surgical clinics (face-to-face and telephone) were the most common encounter, occurring 137 times, median 3 (IQR=1–4) encounters per patient (from first clinic up to surgery date). Due to complexity of symptoms, three patients were reviewed by others specialist teams prior to surgical intervention.

A mean percentage of agreement of the encounters present in EHR with the process map Version 2 was 90% (SD=16). This was calculated by taking the number of encounters that correctly matched the sequence in the process map, divided by the total number of encounters.

Key timepoints were identified following the recording of the presence or absence on an event. Quantitative validation identified several key timepoints where >80% of patients were present, these were surgical clinic, anaesthetic preoperative assessment and admission for surgery (see table 2).

**Table 2:** Key timepoints

Encounters	Number of patients (n=50)*
Initial neurosurgical clinic	44 (88%)
Combined initial clinic and consent	6 (12%)
Surgical consent clinic	39 (78%)
Anaesthetic preoperative assessment	48 (96%)
Admission for surgery	50 (100%)

*n(%); median (IQR)

Although a high percentage of the patient cohort followed the process map, there were some discrepancies. We observed that in six (12%) patients, a combined initial clinic and consent clinic was used, instead of separate clinic encounters. This resulted in patients being seen only once by a neurosurgeon, prior to their admission for surgery. In addition, that five (10%) patients did not have a dedicated consent clinic prior to hospital admission and were instead consented on the day of surgery. Also, we observed that in two cases patients did not attend their anaesthetic assessment.

### Use of educational resources

The educational resources present in version 2 of the process map were also investigated by prospectively surveying 25 patients who had undergone elective spine surgery. The cohort of patients comprised of 17 (68%) males and a median age of 59 (53–71) years of age.

Further investigation of the educational resources identified that the most used resource was verbal information from the surgeon (n=25, 100%), during clinics (n=15, 60%) and on admission for surgery (n=15, 60%). Patients also used written information from their surgeon (n=13, 52%), most often from the clinic letter (n=10, 40%). Patients also received verbal information about their surgery from other healthcare professionals such as clinical nurse specialists, physiotherapist and general practitioners (n=13, 52%). A substantial proportion of patients used electronic resources to learn about their surgery, including self-directed internet search (n=9, 36%), patient education websites (n=7, 28%) and social media (n=2, 8%). Printed leaflet resources were less frequently used (see table 3).

**Table 3:** Educational resources used

Educational resource	n=25*
Written information from surgeon	13 (52%)
Clinic	10 (40%)
Consent clinic	6 (24%)
Verbal information from surgeon	25 (100%)
Clinic	15 (60%)
Consent clinic	12 (48%)
Admission for surgery	15 (60%)
Verbal information from GP or other HCP	13 (52%)
Verbal information from friends and family	9 (36%)
Verbal information from charities/support groups	1 (4.0%)
Verbal information other	1 (4.0%)
Printed hospital resources	7 (28%)
Printed BASS resources	5 (20%)
Printed other	1 (4.0%)
E-learning patient website	7 (28%)
E-learning self-directed internet search	9 (36%)
E-learning journal	0 (0%)
E-learning social media (Twitter, Facebook etc.)	2 (8.0%)
Multimedia	1 (4.0%)

*Median (IQR); n (%).

BASS, British Association of Spine Surgeons; GP, general practitioner; HCP, healthcare professional.

## Discussion

### Principal findings

This is the first application of process mapping to produce a validated pathway for patients awaiting elective spine surgery. This study used process mapping to describe the current patient pathway of patients undergoing elective spine procedures and used a patient questionnaire to identify educational resources commonly used by patients. In addition, process mapping identified key timepoints along the pathway which could be targeted for future quality improvement interventions and automated data collection. The cohort used in this study is heterogenous at a large surgical centre and therefore reflects a typical caseload of patients awaiting elective spine surgery.

First, we generated a process map employing a methodology previously outlined by Antonacci *et al* and subsequently enhanced by Hanrahan *et al*, enabling the creation of a precise depiction of the clinical pathway.^10 12^ This methodology is centred around interviewing members of a multidisciplinary team (MDT) and analysing the results.^11^ It is low cost, time efficient and has multiple clinical benefits by breaking down the complexity of healthcare processes to provide opportunities for quality improvement. It is a methodology adapted from the engineering industry, but we have demonstrated elective spine surgery is a valid use case.

Second, using a patient questionnaire developed from a scoping review by Atlas *et al*,^15^ we identified that the major resource that patients use to learn about their spinal procedure prior to surgery is information from their surgeon, either verbally or through clinic letters. The timepoint that patients reported they learnt most of the information about an upcoming surgery was during surgical clinics. This highlights the importance of clear communication between a patient and their surgeon as it is the main way in which information about a procedure is learnt. Interestingly, 40% of patients used self-directed internet searches or social media to learn about a surgical procedure. The widespread access to the internet has caused a steep increase in the number of patients using the internet to learn about their procedure.^17^ The patients who seek online health information are younger and from higher socioeconomic classes.^17^ Although access to readily available information on the internet empowers patients, it is crucial that patients are guided to trusted resources. It must be recognised that the internet is unregulated and as such can be a source of misinformation. Clinicians should counteract this by providing guidance on trusted resources as it has been shown to positively impact medical decision making and reduce frustration in searching for resources.^17 18^ Our findings indicate that online resources are an important adjunct for clinicians to use when educating patients on spinal procedures.

Third, we found that despite the wealth of information available to patients, many continue to learn from friends and family. Lower back pain is highly prevalent globally^19^ and therefore, it is common for people to know of someone who has had spinal surgery. While surgeons are known to provide consistent, extensive information about operative details and in-patient surgical risks, they provide less information on the impact to quality-of-life surgery can have.^20^ It is perhaps unsurprising that patients seek further information and advice from friends and family, who have a lived experience of a proposed procedure, outside the time limitations of a surgical clinic. In addition, it cannot be overestimated the influence that friends and family members have on a patient’s decision-making to undergo elective surgery.^21^ Two distinct roles are as an information broker, collating sources of information for a patient who may find details of an upcoming surgery overwhelming and patient advocacy, by standing up for patient’s values during clinic when the patient may not feel they have a voice.^21^ Our finding can be used to inform clinicians to involve close friends and family members in the consent process when practically feasible.

Finally, through quantitative validation of the process map, we found that the surgical clinic, anaesthetic preoperative assessment and admission for surgery were key timepoints preoperatively. These timepoints are promising targets for automated data collection which would have numerous benefits in spine surgery research. Currently, the British Spinal Registry is used as a national database, to collect large volumes of clinical and outcome data of patients undergoing spinal surgery in the UK.^22^ However, data is uploaded manually by surgeons, creating an admin workload burden for clinicians. Key timepoints identified in this study are potential critical datapoints automated data collection to facilitate more comprehensive data collection and closer multicentre research collaboration through the enhanced sharing of data. Highlighting key timepoints also gives a target for quality improvement projects to advance the information giving provided to patients. This study identified that the surgical clinic is not only a key timepoint, but also the verbal and written information during surgical clinics are key sources of information that patients use to learn about their upcoming surgery. Since the recall of general information and complications from the consent process remains low,^23^ interventions to improve information recall, targeting the surgical clinics and subsequent written information is advised.

### Findings in the context of the current literature

Lower back pain is the leading cause of years lived with disability in the world, with 266 million people diagnosed each year with degenerative spinal disease.^19 24^ Despite the increasing need for spinal surgery, an ongoing issue is informed consent as it is complex for patients to understand all the material risks from surgery. We have demonstrated in this study that process mapping a spine service is an effective first step towards achieving this goal.

Process mapping has previously been used in healthcare, but the methodological quality of the existing literature varies hugely.^11^ A systematic review of process mapping in healthcare identified that only 45% of process maps were developed in consultation with members of the MDT.^11^ In contrast, our study used mixed methods to develop the process map during a series of semistructured interviews of members of the MDT (stakeholders) and then quantitatively validated it using EHR. In addition, previous studies have used process maps in surgery with an overarching aim of improving efficiency either from a time or financial perspective.^9 25–27^ Chang *et al* used process mapping to illustrate the steps between a patient admission to the emergency department and insertion of an external ventricular drain. This highlighted that access to equipment was a cause of time delay and subsequently, creating a ventriculostomy trolley, significantly reduced time to ventriculostomy.^25^ In spine surgery, Lui *et al* paired lean principles with process mapping to identifying time saving areas for patients undergoing lumbar spinal fusion surgery. The study identified that instrument defects were a cause of 66% of the time delay intraoperatively, a crucial finding in streamlining the efficiency of lumbar fusion procedures.^26^ We have shown that developing a process map for elective spine surgery is a valuable template on which the resources patients use to educate themselves can be illustrated. This will help guide interventions to improve patient education by focusing on key timepoints, where the highest percentage of patients will benefit.

Recently, Hanrahan *et al* has used process mapping to comprehensively define the preoperative, perioperative and postoperative pathways for patients undergoing transsphenoidal surgery.^12^ In the paper, they identified that pituitary ward round entries, pituitary clinical nurse specialist ward round entries and pituitary MDTs were critical data entry points that could be used for automated data entry. Our study builds on this previous work by investigating the educational resources that patients use before spinal surgery. This adds another layer of detail to the process map that provides additional value to the map and has implications for enhancing the provision of information to patients.

### Strengths and limitations

This study demonstrates the use of process mapping to identify the patient pathway prior to surgical intervention in spinal surgery and identifies the educational resources used by patients. This use case for process mapping is novel in the literature but is widely applicable to other healthcare settings. The methodology described is low cost and does not require specialist expertise, and therefore, could be adopted by other healthcare settings primarily as a quality improvement tool. We used a robust methodology from Antonacci *et al* that developed the process map from key stakeholders of the clinical pathway and then later validated the map using real-world patient data.^11^

Although this study prospectively collected data on what educational resources patients used prior to surgery, this cohort was different to the cohort used to validate the process map. Despite both cohorts being representative of elective spinal populations at our centre, the difference may have led to some variation in the process map. There are several limitations of this study that limit the generalisation of the results. Despite the patient cohort being heterogenous for DSD, it was from a single centre and therefore, experiences at other centres may differ. Similarly, validating patients’ presence in a pathway using electronic encounters will not be possible in healthcare systems without EHR. These limitations will be become less significant as the adoption of EHR is increasingly made globally.

## Conclusions

This study used process mapping to define the preoperative patient pathway for patients undergoing elective spine surgery. Surgical clinic, anaesthetic preoperative assessment and surgical admission were the key timepoints identified where over 80% of patients were present. Additionally, using a prospective questionnaire, the study identified that spinal patients mostly use verbal information from the surgeon, during clinics and on admission for surgery to learn about their procedure. These findings can help guide future quality improvement work to enhance patient education prior to surgery.

## Data Availability

Data are available upon reasonable request. Data are available upon reasonable request by contacting the corresponding author.
