# Supplementary material for: Mapping patient education encounters in elective surgery: a cohort study and cross-sectional survey
Source: BMJ Open Qual. 2024 May 27;13(2):e002810. doi: 10.1136/bmjoq-2024-002810 (PMC11131119; doi:10.1136/bmjoq-2024-002810)
Supplement: Supplementary data [file bmjoq-2024-002810supp001.pdf]

SUPPLEMENT and APPENDIX

Supplement

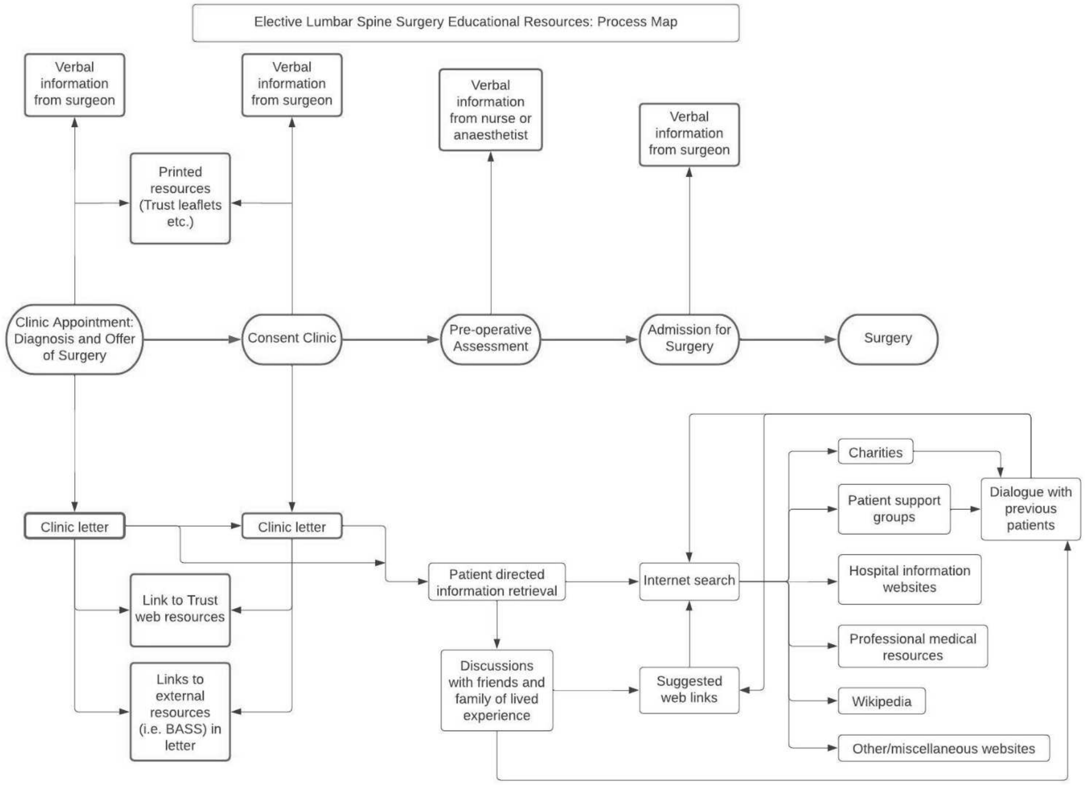

Supplement Figure 1. Process Map Version 1.

Appendix A

Questionnaire

Mapping the clinical pathway for patients undergoing lumbar degenerative spine disease resection

Lead researchers: James Booker + Jack Penn
Email: james.booker.19@ucl.ac.uk
Version 1

Consent to Participate

Study title: Digital workflow analysis of the patient pathway for lumbar degenerative spine disease within the spinal surgery service.

Study aim: Elicit the patient pathway of lumbar degenerative spine disease patients undergoing surgery to target digital interventions.

Confidentiality: Your participation is voluntary and the information you provide, should you wish to do so, will be used for the purpose of refining the content of the assessment tool. Please signal to the researcher any confidential information that you would not want to appear in published form and/or where you wish the source to be made anonymous, please do not hesitate to request so. Should you request anonymity, all identifiable data gathered from this project will remain stored with, and accessible only to the researchers listed.

Authorisation to proceed:

Print name and add date of consent (sign separately, below)

I... Date: ....

In line with the GDPR guidelines that came into force on May 25th, 2018, I hereby agree to:

**Item**	**Initial each that you agree to and leave blank if you do not consent to items**
Be anonymously quoted in publications	
Have my anonymised data used in publications	
Have my anonymised data used in future research	
Be contacted about this research in the future	

Participant signature (giving consent to participate): ....

Process mapping questionnaire

Instructions

This questionnaire asks for information about your professional background and your involvement with the lumbar degenerative spine disease patient pathway for those undergoing surgery. It will take about 5 minutes to complete.

If you do not wish to answer a question, or if a question does not apply to you, you may leave your answer blank.

SECTION A: Professional background

1) What is your primary work area or unit in this hospital?

☐ a. Neurosurgery

☐ b. Ear, nose and throat

☐ c. Radiology

☐ d. Anesthesia

☐ e. Other (please specify) ________________________

2) What is your job title? ____________________

SECTION B: Process experience

1) How many years of experience do you in have in managing this patient group? ____________________

Please indicate your agreement or disagreement with the following statements.

	Strongly Disagree ▼	Disagree ▼	Neither ▼	Agree ▼	Strongly Agree ▼
1. I am routinely involved in the patient pathway of lumbar degenerative spine disease patients undergoing surgery...	☐1	☐2	☐3	☐4	☐5
2. I am directly involved in the patient pathway prior to admission for surgery	☐1	☐2	☐3	☐4	☐5
3. I am directly involved in the patient pathway during their inpatient stay for surgery	☐1	☐2	☐3	☐4	☐5
4. I am directly involved in the patient pathway in the outpatient setting after they have undergone surgery...	☐1	☐2	☐3	☐4	☐5

SECTION C: Initial process map (Please refer to process map now)

The process map presented to you is an initial process map of the patient pathway of lumbar degenerative spine disease patients undergoing surgery at Queen Square from referral to discharge from service. This has been created by two authors based upon their experience.

In this study we want you, as a stakeholder in the process, to help refine this initial process map towards a true representation of the lumbar degenerative spine disease patient pathway. We appreciate there will be nuances, however, this aims to reflect the pathway as accurately as possible.

This will involve you annotating a physical version of the initial process map, adding or removing steps to reflect your perspective on the patient pathway. If there is an aspect of the process you are not involved with, please highlight this to the researcher. The researcher will work with you through the process map and can clarify any queries.

Your input will be collated and synthesised by the research team to develop a refined process map, which aims to be a better representation of the true patient pathway. We will circulate the refined process map back to you with a justification of changes to validate them.

SECTION D: Process map feedback

Thank you for reviewing our process map. Your views will be synthesized with the remaining stakeholder perspectives. The variety of stakeholders include neurosurgeons, clinical nurse specialists, anesthetists, physiotherapists and the MDT coordinator.

Please indicate your agreement or disagreement with the following statements.

	Strongly Disagree ▼	Disagree ▼	Neither ▼	Agree ▼	Strongly Agree ▼
1. The overall process is reflected accurately	☐1	☐2	☐3	☐4	☐5
2. The process from presentation to hospital admission is accurate	☐1	☐2	☐3	☐4	☐5
3. The appropriate variety of stakeholders have been considered for this project (See text above)	☐1	☐2	☐3	☐4	☐5

SECTION E: Proposed interventions

The intended output of this study is to identify mandatory events in the admission of a patient undergoing surgery for a Lumbar degenerative spine disease. We aim to design

digital interventions in Epic (Such as smartphrases) to structure how data is entered at a selection of these mandatory events. We are referring to these interventions as "Structured data entry".

Please indicate your agreement or disagreement with the following statements.

	Strongly Disagree ▼	Disagree ▼	Neither ▼	Agree ▼	Strongly Agree ▼
1. I would be happy to alter my data entry practices supporting structured data entry (e.g. operation note/ward round note template)...........................	☐1	☐2	☐3	☐4	☐5
2. I think structured data entry would improve data quality entered into Epic	☐1	☐2	☐3	☐4	☐5
3. Structured data entry would improve the care of my patients	☐1	☐2	☐3	☐4	☐5
4. Structured data entry would interfere with my current clinical practice	☐1	☐2	☐3	☐4	☐5

SECTION F: Your Comments

Please write any comments you have about research.

THANK YOU FOR COMPLETING THIS SURVEY.

Appendix B

Age:

Gender:

We are looking into resources available to patients undergoing **elective spine surgery** and how they are utilised in **pre-operative patient centred education.**

Which of the following have helped you learn about your spine operation before surgery?
Please tick ☑ all that apply.

Part 1 – Core information sources

1. **Written information from surgeon** ☐

   If you have ticked this box, please indicate when information was provided:

   a) Initial clinic letter ☐ b) Dedicated consent clinic letter ☐

2 **Verbal information from surgeon** ☐

   If you have ticked this box, please indicate when information was provided:

   a) Initial clinic visit ☐ b) Dedicated consent clinic ☐

   c) Admission for surgery ☐

Part 2 - Alternative information sources

1. **Verbal information**

a) GP or other healthcare professional ☐

b) Friends and family ☐

c) Charitable organisations or support groups ☐

d) Other verbal information, please specify: ☐

____

2. Printed information

a) Printed hospital information leaflets ☐

b) British Association of Spine Surgeons leaflets ☐

c) Other written material, please specify: ☐

____

____

3. E-learning

a) Patient education websites e.g., Hospital web resources or British Association of Spine Surgeons ☐

b) Self-directed internet search and general web pages (e.g., YouTube, Wikipedia) ☐

c) Online medical scientific resources (e.g., PubMed scientific journal library) ☐

d) Social media (e.g., Twitter, Facebook) ☐

4. Multimedia educational tools (animated learning module) ☐

Thank you for completing this form.
